# The Prevention of Measles

**Published:** 1898-01-29

**Authors:** 


### THE PREVENTION OF MEASLES.

DR. FRANCIS T. BOND, Gloucester, writes: The writer of the article in the issue of THE HOSPITAL for the 15th inst. appears to object to the "treatment" of this disease being considered a "matter of routine." That must depend very much upon what is to be understood by the term "treatment." I venture to say that the large proportion of cases of measles require treatment of so simple a nature that to any intelligent person who is told what to do and who will do what she is told, it will soon become a question of very ordinary routine indeed. That cases will constantly occur in which skilled medical treatment is necessary no one denies. I expressly recognised the fact. To assume that any amount of "insisting" by medical officers of health will induce the poorer classes to provide medical attendance for all cases of measles is, I venture to say, to show a strange want of acquaintance with the practical aspects of this subject. It may be quite true that a doctor "ought to be called in" whenever a child is attacked with measles, but it is equally certain that in a large number of cases he never will be, and that in many of such cases an intelligent nurse, who has been trained to her work and knows where her duty ends and that of the doctor comes in, will do all that is necessary. It is simply a question whether the poor shall have such help or whether they shall have none. If the writer of the article will be good enough to show how "proper treatment by qualified medical men" is to be ensured in all cases of measles I am quite prepared to abandon my suggestion of trained nurses as a *pis aller*. But until this can be done and the doctors can be properly paid for such work, I doubt whether district medical officers, at any rate, will object to an expedient which will not increase their duties except where there is distinct need for so doing.

⁂ We quite believe that Dr. Bond made his suggestion in all good faith, and that probably many sanitary authorities may be led to agree with him, but that is just the danger of the position. From the lay point of view what he says is very plausible, but it has nothing to do with the right and wrong of the case. The Legislature has instituted a certain definition of a qualified practitioner, and has by law enacted that no unregistered person may hold any appointment as physician, surgeon, "or other medical officer," to any parish, union, or other public establishment, body, or nstitution, or as a medical officer of health. Now we take it that this precludes any public body from appointing an unregistered practitioner, either male or female, for the treatment of disease. Moreover, the General Medical Council, a body appointed by Act of Parliament, having found that some registered practitioners have been in the habit of allowing unqualified persons to "attend or treat patients in respect of matters requiring professional discretion or skill," have declared that "such a substitution of the services of an unqualified person for those of a registered medical practitioner is in its nature fraudulent." The matter is in our mind absolutely clear. Not only from a professional, but also from a legal point of view, the employment of unregistered persons "in respect of matters requiring professional discretion and skill," by any public body or by any registered practitioner, is wrong, while it seems extremely probable that the old woman's notion that the treatment of measles does not require professional discretion and skill is at the bottom of a good deal of the mortality caused by that disease. Sanitary authorities are under no obligation to provide treatment for those suffering from measles, but if they choose to provide it they are under an obligation to employ properly educated and legally qualified persons for the purpose.—ED. *T.H.*

